# Complete Genome Sequence of a Macrolide-Resistant Bordetella pertussis Isolated in Japan

**DOI:** 10.1128/mra.00718-22

**Published:** 2022-09-21

**Authors:** Kentaro Koide, Takahiro Yamaguchi, Chihiro Katsukawa, Nao Otsuka, Tsuyoshi Kenri, Kazunari Kamachi

**Affiliations:** a Department of Bacteriology II, National Institute of Infectious Diseases, Tokyo, Japan; b Division of Microbiology, Osaka Institute of Public Health, Osaka, Japan; University of Maryland School of Medicine

## Abstract

We report the complete genome sequence of macrolide-resistant Bordetella pertussis BP616, which was first isolated in 2018 in Japan. The BP616 genome can serve as a valuable specific reference for genomic and epidemiological studies of this resistant bacterium.

## ANNOUNCEMENT

Bordetella pertussis causes pertussis, commonly referred to as whooping cough. This is a highly contagious respiratory disease that can affect individuals of all age groups. For antibiotic therapy in patients with pertussis, macrolides are the first-choice drugs. However, in the 2010s, macrolide-resistant B. pertussis (MRBP) carrying a A2047G mutation in the 23S rRNA was isolated with increasing frequency in mainland China (1–4). Recently, MRBP has been detected in other Asian countries, such as Vietnam and Japan (5, 6).

In Japan, a MRBP (BP616; alias 2018-52) was isolated for the first time from a 2-month-old boy in Osaka in 2018 (5). Here, we performed whole-genome sequencing of the MRBP isolate BP616. The isolate was cultured on cyclodextrin solid medium at 36°C for 3 days. Genomic DNA was extracted from the colonies using the Genomic-tip 100/G and DNA buffer set (Qiagen, Germany). For long-read sequencing, DNA was sheared using Covaris g-TUBE (Covaris, USA), and a library was constructed using the SMRTbell template prepare kit (Pacific Biosciences, USA). The >17-kb library was then selected with AMPure Beads (Beckman Coulter Inc.), and single-molecule real-time (SMRT) sequencing was performed using the PacBio RSII platform (Pacific Biosciences). A short-read sequencing library was constructed using the TruSeq DNA PCR-Free Kit (Illumina, USA), followed by sequencing on an Illumina NovaSeq 6000 platform with 150 bp paired-end reads. The library preparation and sequencing on both platforms were performed by Macrogen Japan (Tokyo, Japan). A total of 101,593 subreads (*N*_50_ = 14,966 bp) were obtained on the PacBio platform, whereas the Illumina platform yielded 39,652,332 reads. The raw data were filtered using Filtlong version 0.2.0 (https://github.com/rrwick/Filtlong) for long reads (length of >1,000 bp; 10% of poor-quality reads discarded) and fastp version 0.20.1 for short reads (length of >20 bp; sequencing quality score of >20) (7). A hybrid *de novo* assembly using the PacBio and Illumina reads was conducted using Unicycler version 0.4.9 (8), and the resulting genome sequence was annotated using the DFAST pipeline (9). Default parameters were used, except where otherwise noted.

The assembly revealed a single circular chromosome (4,130,169 bp) with a guanine-cytosine content of 67.7%. 3,967 predicted protein-coding sequences, 3 rRNA operons, 1 transfer-mRNA (tmRNA), and 64 tRNAs genes were identified. BP616 has a homogeneous A2047G mutation in each of the three copies of its 23S rRNA gene (corresponding to the A2035G position in its genome). An *in silico* analysis confirmed that BP616 belongs to the genotype MT195 and carries virulence-associated allelic genes (*ptxP1*, *ptxA1*, *prn1*, *fim3A*, and *fhaB3*). The genotype and allelic genes are common in Chinese MRBP isolates (10–12).

The BP616 genome sequence is a high-quality, complete genome sequence for recent MRBP isolates collected in Asia. Given that a non-MRBP genome (vaccine strain Tohama I) has been used as a reference genome in MRBP studies, our BP616 genome sequence can serve as a specific reference for epidemiological studies.

### Data availability.

The complete genome sequence was deposited in DDBJ/EMBL/GenBank under accession number AP024746.1, and the Illumina short-read data were deposited in the DDBJ Sequence Read Archive under DRR300236.
